# Can Biomarkers of Oxidative Stress in Serum Predict Disease Severity in West Nile Virus Infection? A Pilot Study

**DOI:** 10.3390/tropicalmed7090207

**Published:** 2022-08-24

**Authors:** Maxim Van Herreweghe, Annelies Breynaert, Tess De Bruyne, Corneliu Petru Popescu, Simin-Aysel Florescu, Yaniv Lustig, Eli Schwartz, Federico Giovanni Gobbi, Nina Hermans, Ralph Huits

**Affiliations:** 1 NatuRA Research Group, Department of Pharmaceutical Sciences, University of Antwerp, 2610 Wilrijk, Belgium; 2 Department of Adult Infectious and Tropical Diseases, Carola Davila University of Medicine and Pharmacy and Dr Victor Babeș Clinical Hospital of Infectious and Tropical Diseases, 030303 Bucharest, Romania; 3 The Central Virology Laboratory, Public Health Services, Ministry of Health, Sheba Medical Center, Tel Hashomer, Israel & Sackler Faculty of Medicine, Tel Aviv University, Tel Aviv 52621, Israel; 4 Center for Geographic Medicine and Tropical Diseases, Sheba Medical Center, Tel Hashomer & Sackler Faculty of Medicine, Tel Aviv University, Tel Aviv 52621, Israel; 5 IRCCS Ospedale Sacro Cuore Don Calabria, Department of Infectious Tropical Diseases and Microbiology, 37024 Verona, Italy; 6 Institute of Tropical Medicine Antwerp, Department of Clinical Sciences, 2000 Antwerpen, Belgium

**Keywords:** oxidative stress, West Nile virus, West Nile Fever, West Nile Neuroinvasive disease, *Flaviviridae*, biomarkers

## Abstract

West Nile virus (WNV) can cause asymptomatic infection in humans, result in self-limiting febrile illness, or lead to severe West Nile Neuroinvasive disease (WNND). We conducted a pilot study to compare selected biomarkers of oxidative stress in sera of viremic West Nile virus patients and asymptomatic infected blood donors to investigate their potential as predictors of disease severity. We found that total oxidant status was elevated in WNND and in uncomplicated WNV infections (median 9.05 (IQR 8.37 to 9.74) and 7.14 (7.03 to 7.25) µmol H_2_O_2_ equiv./L, respectively) compared to asymptomatic infections (0.11 (0.07 to 0.19) µmol H_2_O_2_ equiv./L) (*p* = 0.048). MDA levels showed a similar trend to TOS, but differences were not significant at α = 0.05. Total antioxidant status did not differ significantly between different disease severity groups. Oxidative stress appears to be associated with more severe disease in WNV-infected patients. Our preliminary findings warrant prospective studies to investigate the correlation of oxidative stress with clinical outcomes and severity of WNV infection.

## 1. Introduction

West Nile virus (WNV) is a single-stranded, positive sense RNA virus part of the genus *Flaviviridae*. It is one of multiple arboviruses of growing public health importance [1]. It is transmitted in bird populations by *Culex* mosquitoes, and several larger mammals, including humans, are considered dead-end hosts. WNV is endemic in the United States and an increasing public health threat across Europe and other continents, mainly due to climate change and global travel [1,2].

Human infections are frequently asymptomatic but lead to West Nile Fever (WNF) in 20% of infected patients, and in approximately 1% of infections to more serious pathologies such as meningitis, encephalitis, or flaccid paralysis [1,3]. Neurological presentations, commonly referred to as West Nile Neuroinvasive Disease (WNND), have fatality rates of approximately 10% [1,4]. Many WNND survivors report significant neurological sequelae that may persist for years after acute infection, including abnormal reflexes, muscle weakness, chronic fatigue, and hearing and sensory loss [1,4]. Age is the main risk factor for severe WNV infection, and WNND incidence increases 1.5-fold with each decade [3].

Our understanding of the pathogenesis, predictors of disease severity and neurological sequelae of neuronal injury in WNV infection remains incomplete. Previous studies implicated oxidative stress (OS), an imbalance between antioxidative defense mechanisms and production of cell-damaging reactive oxygen species (ROS), in *Flavivirus* pathogenesis [5,6,7]. Several studies have observed an increase in ROS in dengue-infected patients by comparing patients’ OS markers to healthy controls [6,7]. However, data about OS in WNV infected patients are scarce. We hypothesized that OS plays an important role in the pathogenesis and severity of WNV disease [8,9]. In this pilot study, we evaluated selected OS biomarkers on a panel of stored human sera from patients with West Nile virus infection of varying severity.

## 2. Materials and Methods

The study protocol was approved by the relevant ethics committees (see ethics statement) and informed consent was provided by all participants. We obtained WNV-RNA qRT-PCR-positive serum samples from blood donors and patients with varying disease severity, i.e., asymptomatic (AS), WNF or WNND, at Sheba Medical Center, Tel Hashomer, Israel, during the 2020 transmission season. The qRT-PCR used primers and probes that targeted the envelope and capsid protein coding regions and the 5′ untranslated region, as described elsewhere [10,11]. Disease severity was assessed by the attending physicians. Patients were classified as WNF when febrile (temperature ≥ 38 °C), presenting with headache, rash or myalgia, and no signs of WNND. Patients were classified as WNND when they presented with encephalitis (altered mental status lasting ≥ 24 h with no alternative cause identified, generalized or partial seizures, new onset of focal neurologic findings, MRI abnormalities suggestive of encephalitis), meningitis (fever and headache AND/OR signs of meningeal irritation), or flaccid paralysis (as acute onset of progressive limb weakness over 48 h AND without sensory abnormalities). AS viremic individuals were identified among blood donors via Magen David Alom, during routine safety evaluation by the central virology lab at Sheba Medical Centre. All blood donors in Israel are screened for WNV RNA during the WNV transmission season using the Procleix WNV assay, a nucleic acid test (NAT) performed on the Procleix Panther system (Grifols, Spain) [12]. Positive NAT results are then confirmed using qRT-PCR (as mentioned above). Samples were collected at one time point only, handled, frozen and stored on-site until shipment to and analysis in the NatuRA lab in Antwerp in 2021. All OS markers were analyzed using commercially available kits, per manufacturer’s instructions: Total Oxidant Status (TOS) and Total Antioxidant Status (TAS) using colorimetric kits (RelAssay Diagnostics, Şehitkamil, Turkey), malondialdehyde (MDA) was quantified using ELISA (My Biosource, San Diego, CA, USA).

SPSS Statistics (IBM; Armonk, NY, USA) was used for statistical analyses. The association of disease severity and markers of OS was analyzed using Kruskal–Wallis tests. All hypotheses were tested at significance level α = 0.05.

## 3. Results

Eight patients were recruited, six of which (75%) were female. Cycle-threshold values (Ct-values) for WNV RNA by qRT-PCR ranged from 32.63 to 41.25. Patients were categorized based on disease severity: four were asymptomatic (age range 18 to 59 years old), two had WNF (aged 23 and 43 years old), and two developed WNND (11 and 84 years old). Patient characteristics, WNV viral loads and serum concentrations of OS markers are shown in Table 1.

**Figure 1:**
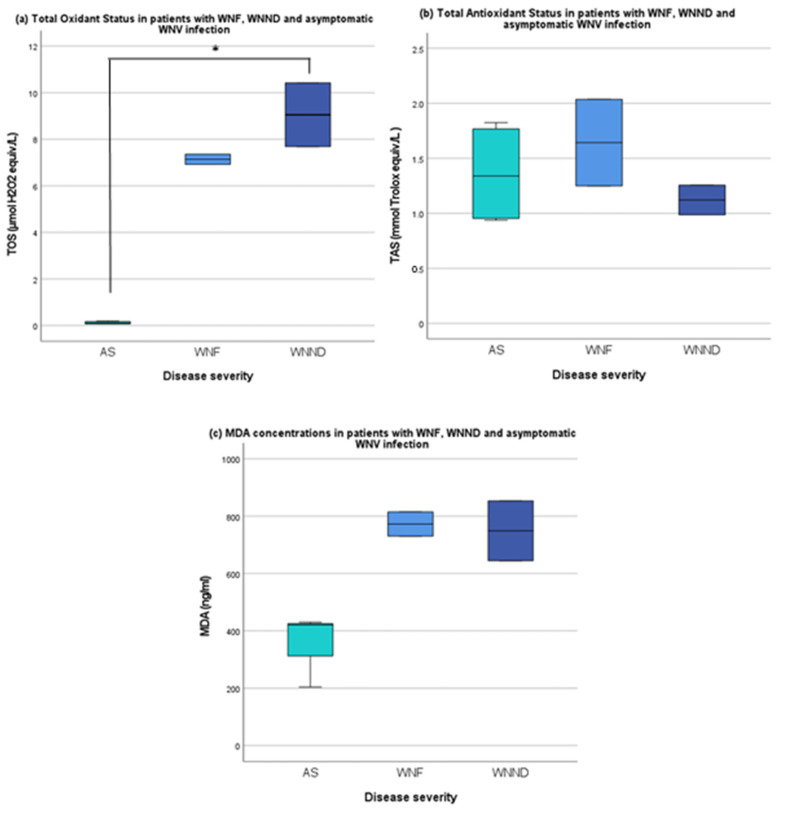
Boxplots of TOS (**a**), TAS (**b**) and MDA levels (**c**) in WNV-infected patients from Israel, per disease severity subgroup. Legend. (**a**) TOS (median) 9.1, IQR [8.4–9.7], 7.1 [7.0–7.3] and 0.1 [0.1–0.12] μmol H_2_O_2_ equiv./L in WNND, WNF and AS groups, respectively. TOS was significantly higher in the WNND and WNF groups (*p* = 0.048) compared to AS. (**b**) TAS (median) 1.1 [1.1–1.2], 1.6 [1.5–1.8] and 1.3 [1.0–1.8] mmol Trolox equiv./L, respectively. TAS was lower in the WNND patients (not significant). (**c**) MDA concentrations were higher in WNND (median) 749.0 ng/mL [696.7–801.3] and in WNF 772.7 [751.5–793.8] ng/mL than in AS (420.3 [312.2–425.0] ng/mL. (not significant, *p* = 0.105). AS = asymptomatic, WNF = West Nile Fever, WNND = West Nile Neuroinvasive Disease, TOS = Total Oxidant Status, H_2_O_2_ = hydrogen peroxide, MDA = malondialdehde, TAS = Total Antioxidant Status. * = *p* < 0.05 (Kruskal–Wallis test).

TOS in the WNND and WNF patients was significantly higher than in AS; 9.05 (8.37 to 9.74) (median, IQR) and 7.14 (7.03 to 7.25) and 0.11 (0.07 to 0.19) µmol H_2_O_2_ equiv./L, respectively (*p* = 0.048) (Figure 1a). Median TAS was 1.12 (1.05 to 1.19), 1.64 (1.45 to 1.84) and 1.34 (0.95 to 1.80) mmol Trolox equiv./L, respectively (Figure 1b). TAS was slightly lower in the two WNND patients, but differences were less pronounced than with TOS or MDA. MDA concentrations were 749.04 (696.74 to 801.33) ng/mL in WNND, 772.65 (751.47 to 793.82) ng/mL in WNF and 420.26 (312.23 to 425.01) ng/mL in the AS group. While MDA levels showed a similar trend to TOS, differences were not statistically significant (*p* = 0.105) (Figure 1c).

## 4. Discussion

We observed a significant increase in TOS in WNF and WNND compared to AS. The increased TOS, a summary parameter of the total oxidative capacity in a sample, suggests that oxidation is more prominent in WNF and WNND than in AS [13]. Because of the very small sample size, we were not able to discriminate between WNF and WNND using TOS in this pilot study. However, using a larger study population and parametric tests, a significant difference in TOS between WNF and WNND might still be observable. Still, the marked difference in TOS between symptomatic WNV disease and AS individuals calls for a detailed assessment of the redox status in WNV infections and an investigation of more specific biomarkers than TOS as a potential predictor of severe WNV disease.

Whereas TOS does not necessarily reflect oxidative damage (OD) to the host, MDA, the most widely studied end-product of lipid peroxidation, is a highly relevant marker of OD in neuronal toxicity studies [7,14]. The observed histopathology of the brain in WNND resembles that of neurodegenerative diseases, where brain cell damage results from lipid peroxidation [9,15]. This similarity may indicate that WNV-induced OS is an important contributor to the observed brain injury in WNND. We observed higher MDA levels in symptomatic patients, suggesting that lipid peroxidation was increased, although not significant, at α = 0.05. Therefore, our pilot study does not allow us to conclude that disease severity is positively correlated with the extent of OD to the host.

Studies of more specific biomarkers of OS than TOS in dengue virus (another member of the *Flavivirus* genus) infections detected significant differences in subgroups with varying disease severities. Protein carbonylation (PCO) was higher in dengue hemorrhagic fever (DHF) than in dengue fever in two studies by Soundravally et al. [16,17]. Patra et al. found higher intracellular ROS levels in erythrocytes of severely ill dengue patients than in patients with mild disease [18].

TAS levels did not follow the same distinctive trends as TOS and MDA, as shown in Figure 1. In other studies, TAS was significantly lower in patients with DHF and dengue shock syndrome than in those with uncomplicated dengue, but only later in the course of infection (day 5 and 7 after symptom onset) [16]. While TAS summarizes the total capacity of the antioxidant defense against ROS, TAS levels are more difficult to interpret because of a variety of confounders, such as dietary antioxidant intake and lifestyle [13]. Our results may indicate that the antioxidant capacity across different groups is comparable, but the investigation into the relationship between TAS and disease severity requires further study. Prospective, controlled studies are needed to establish if there is a correlation between the age-related declining efficiency of endogenous antioxidant systems and increased susceptibility to WNV-induced oxidative stress [19,20].

Because we studied single samples only, no changes in OS biomarkers over time could be established within the different severity subgroups. Longitudinal follow-up of WNV-infected patients is needed to study the intra-host kinetics of these biomarkers. Only markers that differentiate between patients at risk for WNND and patients with mild disease in the early stages of infection are likely to have diagnostic utility for clinical management. We do know that all patients here were in the acute stage of infection during sampling, as shown by their Ct values. These values are indicative of acute WNV infection in serum: in a paper from Lustig et al., WNV viral load in serum was shown to be less than 100 copies/mL on average [12]. A low viral load in the sample leads to high Ct values. Ct values in serum, plasma or cerebrospinal fluid of WNV-infected patients are almost always higher than 30, due to the low levels or even absence of viraemia at the time of symptom onset [12].

Important limitations apply to our study. First and foremost, the small sample size seriously limited statistical analysis. The limited number of subjects required the use of non-parametric tests, with less power compared to parametric testing. This makes it difficult to detect significant differences between the different disease severity groups. Second, while all samples were stored at −80 °C immediately after being received in the lab, OS marker measurements are susceptible to pre-analytical variations, and we cannot exclude this to have occurred in our pilot study. Third, variations in the duration of infection at the time of sampling could account for the observed differences in biomarker assays between individual patients. Nonetheless, all patients were in the acute, viremic stage of WNV infection, with viral loads in a similar range (Ct values 32.6–41.3). This duration could affect biomarker measurements. Still, we think these pilot results warrant further research into using more selective biomarkers for OS as potential predictors, reflecting its role in the neurological symptoms seen in WNF/WNND, and to see whether this OS could lie at the base of the observed pathology.

## 5. Conclusions

In our pilot study, we observed higher TOS levels in WNF and WNND patients than in asymptomatic WNV-infected controls. Prospective studies are warranted to investigate the association of increased lipid peroxidation and declining total antioxidant capacity with disease severity in WNV infections. We are planning an adequately powered prospective controlled cohort study to evaluate the clinical relevance of an extensive panel of peripheral OS biomarkers as predictors of disease severity in patients with WNV and related *Flavivirus* infections. In the projected study, we will also look at intra-host kinetics of the selected OS biomarkers at pre-specified timepoints since symptom onset. The identification of OS biomarkers as predictors of disease severity and long-term outcomes of WNND may assist physicians in the clinical management of patients with WNV infection.

## Figures and Tables

**Table 1:** Serum concentrations of markers of oxidative stress in 8 WNV-infected patients from Israel.

Patient	Age	Sex	WNV (Ct-Value)	Disease Severity	TOS (µmol H_2_O_2_ Equiv./L)	TAS (mmol Trolox Equiv./L)	MDA (ng/mL)
1	84	female	32.63	WNND	10.42	1.26	644.44
2	11	female	41.25	WNND	7.69	0.99	853.63
3	23	female	38.38	WNF	7.36	2.04	730.30
4	43	female	33.13	WNF	6.92	1.25	814.99
5	59	female	36.64	AS	0.07	1.71	204.19
6	21	female	35.29	AS	0.07	0.97	^a^
7	18	male	35.58	AS	0.20	1.83	420.26
8	20	male	37.50	AS	0.15	0.94	429.76

Legend. WNV = West Nile virus, Ct-value = cycle threshold value, WNND = West Nile Neuroinvasive Disease, WNF = West Nile Fever, AS = asymptomatic, TOS = Total Oxidant Status, H_2_O_2_ = hydrogen peroxide, TAS = Total Antioxidant Status, MDA = malondialdehyde, ^a^ = measurement omitted due to spurious results. Each value for TOS, TAS and MDA is the mean of 2 measurements.

## Data Availability

All data generated or analyzed during this study are included in this published article.
